# Extreme Community PrEP Stigma Perceptions as a Potential Deterrent to PrEP Use Among Black and Latino Men Who Have Sex with Men in the Deep South

**DOI:** 10.1007/s10461-025-04977-4

**Published:** 2025-12-05

**Authors:** John Guigayoma, Dennis H. Li, DeMarc Hickson, Mariano Kanamori, Tyler Wray

**Affiliations:** 1 Impact Institute, Northwestern University, Chicago, IL USA; 2 Department of Psychiatry and Behavioral Sciences, Northwestern University Feinberg School of Medicine, Chicago, IL USA; 3 Us Helping Us, People Into Living, Inc., Washington, DC USA; 4 Department of Public Health Sciences, Miller School of Medicine, University of Miami, Miami, FL USA; 5 Department of Behavioral and Social Sciences, Brown University School of Public Health, Providence, RI USA

**Keywords:** Men who have sex with men, PrEP stigma, PrEP use, Black/African American, Hispanic/Latino

## Abstract

Black and Latino men who have sex with men (MSM) in the Deep South have the lowest HIV pre-exposure prophylaxis (PrEP) use rates in the United States, and PrEP stigma may deter PrEP use. However, most research on PrEP stigma is at the interpersonal level, which hinders the development of community-level PrEP anti-stigma campaigns. To address this knowledge gap, we conducted a secondary analysis of an online survey of Black and Latino MSM in the Deep South who are not living with HIV (*n* = 281). Multinomial logistic regression models were used to assess associations between the Community PrEP-Related Stigma Scale (Community-PSS), its four subscales, and PrEP use (never, former, current), controlling for covariates. We found no evidence of an association between the overall Community-PSS nor three of the four subscales and the likelihood of never PrEP use versus current PrEP use. However, we found that a 1-point increase in the extreme stigma perception subscale (i.e., views that community members believe PrEP users are living with HIV, bad people, or hiding something) was associated with a 16% higher relative risk of never PrEP use versus current PrEP use (*p* = .019, 95% CI: 1.03–1.32). We also found no evidence of a relationship between Community-PSS nor its subscales and the likelihood of former PrEP use versus current PrEP use. Given these results, extreme stigma perceptions may deter current PrEP use among Black and Latino MSM populations. PrEP campaigns that depict PrEP users as everyday people may be an effective socio-structural approach to increasing PrEP use.

## Introduction

Black and Latino men who have sex with men (MSM) in the Deep South experience disproportionately high rates of HIV diagnoses [1]. HIV pre-exposure prophylaxis (PrEP) is a highly effective strategy to decrease new HIV infections [2], but PrEP use is disproportionately low among Black and Latino MSM [3–5]. One barrier to PrEP use is PrEP stigma, which can be defined as negative social judgments toward PrEP users that limit access to PrEP or alienate those using PrEP [6, 7]. Previous research has found a negative association between PrEP stigma and current PrEP use among Black and Latino MSM [8–10].

PrEP stigma among Black and Latino MSM in the Deep South has roots within racism and homophobia, which can disproportionately limit PrEP use in these populations. For example, medical mistrust is prevalent among Black MSM in the Deep South and originates from a legacy of race-based mistreatment [11]. Studies have documented how this fear and skepticism of the medical establishment has contributed to lower likelihood of discussing PrEP with a doctor or stigmatizing concerns about PrEP use as a form of government control or as a source of HIV infection from PrEP patients’ friends and family [12, 13]. Other studies report Black and Latino MSM in the region avoiding PrEP based on concerns about being seen as promiscuous or having sex with men [14–17]. This connection between racism, homophobia and PrEP stigma is especially important in the Deep South because racism and homophobia are widely present in the region [18, 19], which in turn could contribute to frequent experiences of PrEP stigma among Black and Latino MSM. For example, one study of baseline data from a prevention trial of 363 Black MSM in the South reported that 54% experienced racism and discrimination and 60% experienced sexual minority stigma [20], which suggests that a large proportion of those with a high need for PrEP may be subject to PrEP stigma via racism and homophobia.

To address PrEP stigma in the Deep South and encourage PrEP use, public health practitioners can develop targeted strategies that diffuse PrEP stigma’s most important underlying beliefs [7]. However, most validated PrEP stigma scales are unidimensional, which obscures a deeper examination of these beliefs. For example, the validation study of the 12-item HIV PrEP Stigma Scale reported that higher PrEP stigma had a negative association with current PrEP use in a national sample of sexual minority men [21]. This scale also performed well in a validation study of Black sexual minority men [22]. However, since a factor analysis indicated a unidimensional structure of this scale, analyses of more specific aspects of PrEP stigma are limited.

In addition, a study using the 10-item PrEP Stigma and Positive Attitudes scale reported that higher PrEP stigma was associated with lower odds of current PrEP use in a national sample of 151 Black sexual minority men [10, 23]. This scale also performed well in a validation study of Latino sexual minority men [24]. This scale has two subscales, one for PrEP stigma and one for positive attitudes toward PrEP use. However, the studies reported results from the full scale alone, which obscures interpretations of potentially important underlying beliefs.

To fully identify the most relevant PrEP stigma beliefs, PrEP stigma studies should use validated measures that isolate key PrEP stigma constructs, thus allowing researchers to evaluate PrEP stigma with the full scale and its component subscales. To address this gap in the literature, we conducted this study using the validated 14-item Community PrEP-Related Stigma Scale. We chose this scale for two reasons. First, this scale has four subscales that could be relevant to understanding specific aspects of PrEP stigma. These subscales consist of three aspects of PrEP stigma that could discourage PrEP use and one set of positive views that could encourage PrEP use: (1) stigma of poor judgment and substance use (PrEP users as irresponsible), (2) extreme stigma perceptions (PrEP users as immoral), (3) stigma of sexual behavior (PrEP users as promiscuous), and (4) positive community perceptions (PrEP users as good) [25]. Second, we chose this scale because it examines stigma at the community level, which contrasts with most PrEP stigma research, which examines stigma at the interpersonal level [26]. By understanding stigma at the community level, findings from this study can better inform community-level anti-stigma initiatives, such as social marketing campaigns.

In addition, relatively limited research exists on PrEP stigma and former PrEP use, and much of the research on this relationship is limited primarily to qualitative studies [27, 28]. For example, one qualitative study of 30 Black sexual minority men in Mississippi on PrEP identified PrEP-related stigma as a challenge to maintaining PrEP use, including fears that family members, church members, or sexual partners may learn that study participants were taking PrEP [27]. One of the few quantitative studies that examined PrEP stigma and former PrEP use using a validated scale found that former PrEP users had lower PrEP stigma than never PrEP users [21]. However, comparisons between former and never PrEP users only provide information on how PrEP stigma may be related to ever starting PrEP, and such analyses provide inadequate information on how PrEP stigma may be related to ending PrEP use. Research on the relationship between PrEP stigma, especially at the community level, and former PrEP use can contribute to the development of public health strategies to maintain PrEP use.

To address these issues, we conducted a secondary analysis of an online survey of Black and Latino MSM across the Deep South [29] to examine the relationships between (1) PrEP stigma and current PrEP use and (2) PrEP stigma and former PrEP user. Given prior research suggesting the negative relationship between PrEP stigma and PrEP use [8–10], we hypothesized the following:

- Higher community PrEP stigma is associated with never using PrEP,
- At least one of the four aspects of community PrEP stigma is associated with never using PrEP,
- Higher community PrEP stigma is associated with being a former PrEP user, and
- At least one of the four aspects of community PrEP stigma is associated with being a former PrEP user.

## Methods

### Recruitment and Procedures

This study is a secondary analysis of data from an online survey of HIV-negative or unknown status Black and Latino MSM across the Deep South to understand their preferences for HIV self-testing programs [29]. Recruitment was embedded within the online campaigns of two national randomized controlled trials of MSM who test infrequently [30, 31]. The campaigns consisted of advertisements on social networking websites such as Facebook and Instagram and online influencer videos on websites such as YouTube and TikTok [32]. Potential participants who clicked an online ad completed a study screener to determine eligibility. Recruitment was from March 2023 to December 2023.

Eligibility criteria consisted of the following: (1) at least 18 years old, (2) cisgender male, transgender (including transmen or transwomen), nonbinary or gender nonconforming, (3) Hispanic/Latino or non-Hispanic/Latino Black, (4) lived in a state in the Southern US, (5) anal or vaginal sex with a man in the past year, (6) never diagnosed with HIV, and (7) fluent in English or Spanish. Eligible individuals provided informed consent via an online consent form and subsequently completed an online survey hosted on Qualtrics (Qualtrics, Provo, Utah). Participants received $25 through a reloadable debit card sent to their home address for completing the online survey. All study procedures received human research ethics approval from the Brown University Institutional Review Board.

Out of an initial sample of 317 participants, 27 (9%) identified as transgender or gender nonconforming. Given the unique barriers to PrEP use in this population [33] and the very low representation in this sample, we limited our analyses to cisgender men only (*n* = 290).

### Measures

*Independent variable: Community PrEP-Related Stigma Scale (Community-PSS)* [25]. This 14-item scale was originally validated with a sample of 108 multiracial and multiethnic men who have sex with men in Florida. The scale had high internal consistency based on Cronbach’s alpha (α = 0.86), and validation assessments showed higher stigma scores were associated with lower PrEP knowledge, lower HIV knowledge, higher HIV PrEP Stigma Scale scores, and higher likelihood of condom usage with a partner on PrEP.

The developers of the scale also conducted a principal component analysis using scree plots and Eigenvalues to identify four subscales. Table 1 presents the four subscales of the Community-PSS, the scale items for each subscale, and the corresponding Cronbach’s alphas for each subscale in the current study. In this sample, the scale had high internal consistency (α = 0.88) and acceptable to high internal consistency for three of the subscales (α = 0.73–0.88). The positive community perception subscale had questionable internal validity (α = 0.68).

**Table 1:** Subscales of the community PrEP-Related stigma scale with corresponding cronbach’s alphas of current study sample (*n* = 281)

Subscales	Survey items (Prompt: People think that people who are on PrEP are …)	Cronbach’s alphas of current study sample
Stigma of poor judgment and substance use	-More likely to engage in sex under the influence of drugs	0.88
	-More likely to have a sexually transmitted infection	
	-Less picky about their sex partners	
	-More likely to use drugs	
	-More likely to abuse alcohol	
	-More likely to cheat on their partner	
Extreme stigma perceptions	-Possibly living with HIV	0.73
	-Usually bad people	
	-Hiding something	
Stigma of sexual behavior	-Having sex with a lot of people	0.87
	-More likely to have sex with strangers	
	-Having riskier sex	
Positive community perception	-Taking responsibility for their health*	0.68
	-Protecting themselves and others*	
Full scale	--	0.89

* Reverse coded

This study used the prompt introducing the Community-PSS as reported in the original manuscript (“People think that people who are on PrEP are”) [25], but did not include the full prompt directing participants to consider the perception of those in their “surrounding community.”

*Dependent variable: PrEP use.* The current study defined PrEP to include all available formulations: “PrEP, pre-exposure prophylaxis, emtricitabine/tenofovir, Truvada^®^, Descovy^®^, Apretude^®^.” The two questions used to code this variable were “Are you currently taking PrEP to keep from getting HIV?” and “Have you ever taken anti-HIV medicines to keep from getting HIV?” Based on combinations of “Yes” or “No” responses, participants were categorized into three groups: current PrEP use, former PrEP use, and never PrEP use.

*Covariates*: The survey included several socio-demographic characteristics that have been empirically shown or are theoretically related to PrEP use, such as demographic factors (including race/ethnicity, age, gender identity, education, and income); cognitive factors (HIV risk perception [34], HIV stigma [35], resilience [36], depression [37]); social factors (social support [38], medical mistrust [39], patient activation [40], everyday discrimination [41]); and two measures of perceived PrEP social norms (percentage of friends/peers on PrEP, percentage of all gay and bisexual men on PrEP) [42].

### Statistical Analyses

We conducted bivariate analyses to assess potential differences between PrEP use categories and potential covariates with chi-square tests for proportions and ANOVA tests for means. Given the three levels of PrEP use, we used multinomial regression models to estimate relative risk ratios; we fit separate models for the overall Community-PSS and its four subscales. All covariates associated with PrEP use in bivariate analyses were included in our final regression models. For models with statistically significant relationships between the measure of community PrEP stigma and PrEP use, we planned to also calculate and graph predicted probabilities to understand potential trends in PrEP use. Listwise deletion was used to address missing data because the prevalence of missing data for each variable in the final sample was less than 5%. Statistical significance across all analyses was set at *p* <.05. We used Stata SE 18 (StataCorp LLC, College Station, TX) for all analyses.

## Results

Out of the 290 responses in the original analytical sample, we excluded 9 participants (3%) who did not complete the Community-PSS, resulting in a final analytical sample of 281 participants (Table 2). Of these participants, 131 (47%) were non-Hispanic Black and 150 (53%) were Hispanic/Latino. This sample consisted of 124 current PrEP users (44%), 38 former PrEP users (14%), and 119 never PrEP users (42%). In bivariate analyses (Table 2), PrEP use was associated with education, HIV risk perception, depression, and both measures of PrEP social norms. We adjusted for these variables in all statistical models.

**Table 2:** Characteristics of black and Latino men who have sex with men in the deep South by PrEP use (*n* = 281)

Characteristics	Current PrEP use *n* = 124 (%)	Former PrEP use *n* = 38 (%)	Never PrEP use *n* = 119 (%)	*p*-values
Race/ethnicity				0.493
Non-Hispanic Black	55 (44.35)	15 (39.47)	61 (51.26)	
Hispanic/Latino	69 (55.65)	23 (60.53)	58 (48.74)	
Language preference				0.543
English	119 (95.97)	37 (97.37)	117 (98.32)	
Spanish	5 (4.03)	1 (2.63)	2 (1.68)	
Age				0.185
< 25	13 (10.48)	7 (18.42)	15 (12.61)	
≥ 25	111 (89.52)	31 (81.58)	104 (87.39)	
Sexual identity				0.158
Gay/homosexual	105 (84.68)	34 (89.47)	90 (75.63)	
Bisexual	16 (12.90)	3 (7.89)	20 (16.81)	
Straight/other	3 (2.42)	1 (2.63)	9 (7.56)	
Education				< 0.001*
Less than college degree	23 (18.55)	10 (26.32)	53 (44.54)	
College degree or higher	101 (81.45)	28 (73.68)	65 (54.62)	
Missing	0 (0)	0 (0)	1 (0.84)	
Rural ZIP code				0.466
No	121 (97.58)	36 (94.74)	117 (98.32)	
Yes	3 (2.42)	2 (5.26)	2 (1.68)	
Income				0.328
<$30,000	19 (15.32)	9 (23.68)	26 (21.85)	
≥$30,000	105 (84.68)	29 (76.32)	93 (78.15)	
Employment				0.205
Full-time	89 (71.77)	27 (71.05)	71 (59.66)	
Part-time	10 (8.06)	2 (5.26)	16 (13.45)	
Unemployed	10 (8.06)	4 (10.53)	20 (16.81)	
Other	15 (12.10)	5 (13.16)	12 (10.08)	
Current health insurance				0.592
Yes	107 (86.29)	34 (89.47)	99 (83.19)	
No	17(13.71)	4 (10.53)	20 (16.81)	
HIV risk perception	25.58 (0.62)	29.18 (1.12)	26.27 (0.63)	0.020*
HIV stigma	29.34 (0.69)	30.13 (1.23)	30.13 (0.71)	0.693
Social support	22.14 (0.50)	20.55 (0.89)	20.63 (0.51)	0.073
Patient activation	71.20 (1.75)	68.47 (3.17)	68.52 (1.80)	0.520
Depression	20.03 (0.56)	23.11 (1.00)	21.36 (0.57)	0.021*
Medical mistrust	2.48 (0.05)	2.67 (0.10)	2.58 (0.06)	0.175
Everyday discrimination	22.32 (0.82)	22.13 (1.47)	21.92 (0.83)	0.942
Resilience	2.46 (0.07)	2.56 (0.13)	2.60 (0.07)	0.382
PrEP social norms (% of friends/peers on PrEP)	50.39 (2.48)	37.71 (4.46)	29.21 (2.63)	< 0.001*
PrEP social norms (% of gay/bisexual men on PrEP)	49.40 (1.94)	51.87 (3.49)	40.57 (1.99)	0.002*
Community PrEP-related stigma	36.29 (0.86)	37.74 (1.55)	37.85 (0.88)	0.411

*p <.05

We found no evidence of a relationship between never PrEP use and the overall Community-PSS scale (RRR = 1.02, *p* =.165, 95% CI: 0.99–1.06) nor did we find evidence of a relationship between never PrEP use and three of the four subscales (RRRs ranging from 0.96 to 1.13, p-values ranging from 0.145 to 0.407). However, we found that a 1-point increase in the extreme stigma perception subscale was associated with a 16% higher relative risk of never PrEP use compared to current PrEP use (RRR = 1.16, *p* =.019, 95% CI: 1.03–1.32).

In addition, we found no evidence of a relationship between former PrEP use and the overall Community-PSS scale (RRR = 1.01, *p* =.692, 95% CI: 0.97, 1.05) nor did we find evidence of a relationship between former PrEP use and any of the four subscales (RRRs ranging from 0.89 to 1.05, p-values ranging from 0.431 to 0.692).

**Fig. 1:**
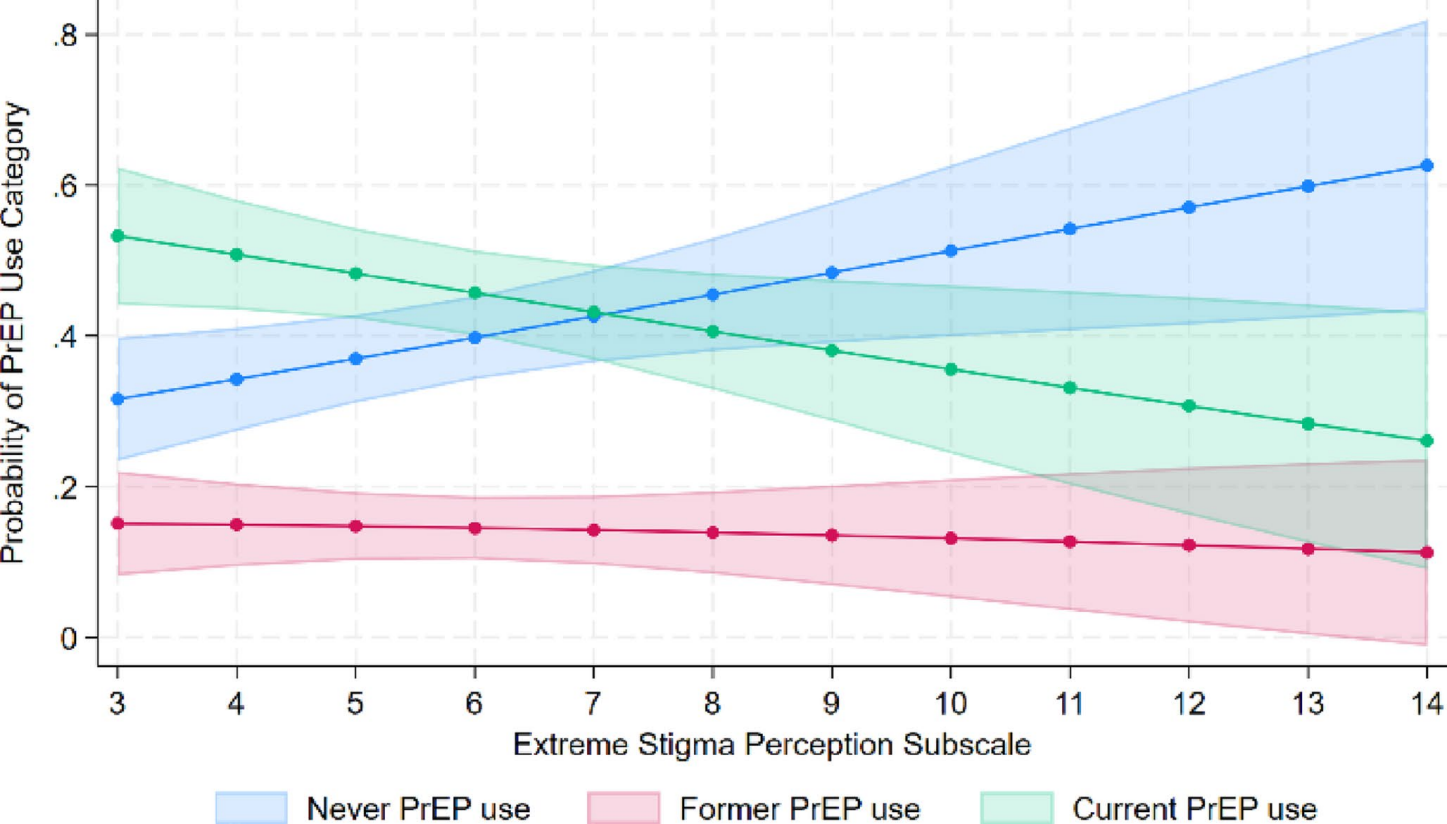
Community PrEP-Related Stigma Scale subscale 2 and PrEP use. Higher scores indicate higher perceived extreme community PrEP stigma

Figure 1 presents the predicted probabilities of each PrEP use category across the range of extreme stigma perception subscale scores. These trends indicate that increases in the extreme stigma perception subscale correspond with both a decrease in the probability of current PrEP use and an increase in the probability of being a never PrEP user. Notably, we found no evidence of change in trend in the probability of former PrEP use.

## Discussion

This secondary analysis of an online survey of Black and Latino MSM in the Deep South sought to address the relationship between community PrEP stigma and PrEP use in these populations. Taken as a whole, this analysis suggests that a broad measure of community PrEP stigma as measured by the Community-PSS may not adequately describe the effect of community PrEP stigma on PrEP use. Instead, research should focus on examining extreme stigma perception (i.e., views that community members believe that PrEP users are living with HIV, bad people, or hiding something) as a specific potential deterrent to PrEP use.

Our finding that higher scores on the extreme stigma perception subscale may limit current PrEP use advances previous PrEP stigma research beyond the more frequently examined PrEP stigma belief among Black and Latino MSM that PrEP users are promiscuous [7]. Our findings may help explain the limited uptake of PrEP despite the existence of campaigns that seek to overturn this specific belief. For example, the PrEP4Love campaign in Chicago consisted of social media advertisements and public transportation ads that featured sex-positive imagery and slogans (“Spread tingle” “Catch desire”) with Black sexual and gender minority couples [43]. Although exposure to this campaign was associated with PrEP use in a community survey [43], a longitudinal study that randomized a small sample (*n* = 96) of Black sexual minority men to PrEP4Love and two other PrEP anti-stigma campaigns with non-sexual positive messaging found no difference in PrEP use outcomes across the three campaigns [44]. Our findings further confirm that public health campaigns that use sex-positive messaging to overturn the stigmatizing belief of PrEP users as promiscuous may miss the mark among Black and Latino MSM, particularly in the Deep South.

Instead, community-level PrEP anti-stigma initiatives that show PrEP users as everyday people may be more effective than focusing on PrEP users’ sex lives alone. Such campaigns can show PrEP users engaged in daily life, such as at work or school and with friends or family. Findings from this study can also inform the design of other interventions to promote PrEP. For example, social network interventions can recruit trusted opinion leaders to model PrEP use as a routine health practice [45, 46]. Given the strong valence of the items within the extreme stigma perception subscale, strategies can also include practices to discretely access PrEP. These strategies include integrating PrEP care into primary care settings or pharmacies as opposed to requiring access through specialists or LGBT health organizations [47, 48]. Tele-PrEP services can also provide PrEP care from the privacy of one’s home [49]. Future research can evaluate which strategies may most effectively overcome extreme stigma perceptions to increase PrEP use in these populations. The finding that overall community PrEP stigma had no evidence of a relationship to never PrEP use contrasts with previous studies of PrEP stigma among MSM in the US that report higher PrEP stigma associated with lower PrEP use [8–10]. Results from this study may differ from previous research because those studies examined PrEP stigma at more personal levels via internalized stigma [9], anticipated stigma [8], and injunctive norms [10], while Community-PSS examines PrEP stigma at a broader community level. As such, these results suggest that more personal forms of PrEP stigma, such as the possibility of direct judgement from one’s peers or the immediate need to conceal PrEP use from others, might more directly deter PrEP use than overall negative perceptions of PrEP use in one’s community. Future studies can analyze PrEP stigma with other validated measures and their component subscales to identify the most relevant aspects of PrEP stigma on PrEP use.

Interestingly, this analysis also found no evidence of a relationship between community PrEP stigma and former PrEP use. These results contribute to the limited research on PrEP stigma and becoming a former PrEP user, suggesting that community PrEP stigma may not affect PrEP engagement among Black and Latino MSM in the Deep South. One potential reason for these results is that those who start PrEP may be able to overcome community PrEP stigma to begin with and may stop using PrEP for other reasons. These other reasons may include structural factors such as health insurance and housing access, concerns with side-effects, and changes in risk behavior [50, 51]. Future studies can also use other validated measures and their component subscales to analyze the relationship between PrEP stigma and former PrEP use.

This study is subject to several limitations. First, this online convenience sample of Black and Latino MSM across the Deep South has low representation of participants in rural areas (*n* = 7, 2%), who prefer Spanish language (*n* = 8, 3%), or who are transgender or gender non-conforming (*n* = 27 in the original sample of 317 participants, 9%). These groups experience greater disparities to PrEP use in the Deep South [52–54], and future studies should examine PrEP stigma with larger samples of these populations. Second, although Community-PSS is a validated measure among sexual minority men in a Southern state (Florida) [25], the original sample used to validate this tool consisted of only 37 Hispanic (35%) and 7 non-Hispanic Black (7%) participants. However, findings from this study of the negative relationship between a Community-PSS subscale and PrEP use coincide with other studies of PrEP stigma and PrEP use among Black sexual minority men [10].

Lastly, since this study used only a partial prompt introducing the Community-PSS (“People think that people who are on PrEP are”) [25], this may introduce some noise into the measure based on differing interpretations of the prompt. However, Cronbach’s alphas for Community-PSS and its subscales indicate acceptable-to-high internal consistency across nearly all measures, suggesting that participants had consistently similar interpretations of the survey tool.

This study provides evidence that perceptions of extreme PrEP stigma in one’s community may hinder PrEP use among Black and Latino MSM in the Deep South. Given these findings, public health campaigns and other interventions that work to overturn these specific stigmatizing perceptions across the region may contribute to increased PrEP use in these populations. By increasing PrEP use among Black and Latino MSM, public health efforts can further work to minimizing one of the largest racial/ethnic disparities in HIV diagnosis in the US.
